# Association between Ocular Sensory Dominance and Refractive Error Asymmetry

**DOI:** 10.1371/journal.pone.0136222

**Published:** 2015-08-21

**Authors:** Feng Jiang, Zheyi Chen, Hua Bi, Edgar Ekure, Binbin Su, Haoran Wu, Yifei Huang, Bin Zhang, Jun Jiang

**Affiliations:** 1 Medical School of Chinese PLA, Beijing, China; 2 Department of Ophthalmology, Jinling Hospital, Nanjing, China; 3 Eye Hospital of Wenzhou Medical University, Wenzhou, China; 4 College of Optometry, Nova Southeastern University, Davie, Florida, United States of America; School of Ophthalmology and Optometry and Eye Hospital, Wenzhou Medical University, Wenzhou, Zhejiang, China., CHINA

## Abstract

**Purpose:**

To investigate the association between ocular sensory dominance and interocular refractive error difference (IRED).

**Methods:**

A total of 219 subjects were recruited. The refractive errors were determined by objective refraction with a fixation target located 6 meters away. 176 subjects were myopic, with 83 being anisometropic (IRED ≥ 0.75 D). 43 subjects were hyperopic, with 22 being anisometropic. Sensory dominance was measured with a continuous flashing technique with the tested eye viewing a Gabor increasing in contrast and the fellow eye viewing a Mondrian noise decreasing in contrast. The log ratio of Mondrian to Gabor’s contrasts was recorded when a subject just detected the tilting direction of the Gabor during each trial. T-test was used to compare the 50 values collected from each eye, and the t-value was used as a subject’s ocular dominance index (ODI) to quantify the degree of ocular dominance. A subject with ODI ≥ 2 (p < 0.05) had clear dominance and the eye with larger mean ratio was the dominant one. Otherwise, a subject had an unclear dominance.

**Results:**

The anisometropic subjects had stronger ocular dominance in comparison to non-anisometropic subjects (rank-sum test, p < 0.01 for both myopic and hyperopic subjects). In anisometropic subjects with clear dominance, the amplitude of the anisometropia was correlated with ODI values (R = 0.42, p < 0.01 in myopic anisometropic subjects; R = 0.62, p < 0.01 in hyperopic anisometropic subjects). Moreover, the dominant eyes were more myopic in myopic anisometropic subjects (sign-test, p < 0.05) and less hyperopic in hyperopic anisometropic subjects (sign-test, p < 0.05).

**Conclusion:**

The degree of ocular sensory dominance is associated with interocular refractive error difference.

## Introduction

Ocular dominance refers to the tendency for the visual system to give more relative preference to the processing of inputs signals from one eye over the other [1–3]. Depending on the operational definition and specific measurements, ocular dominance could be classified into sighting, motor, and sensory dominance. Sighting dominance [4–9] refers to the preferential use of one eye over the fellow eye in fixating a target. It is generally considered to be related to the judgment of visual direction [10, 11], although some studies have shown that such correlation with the judgment of visual direction is not strong [12]. In motor dominance, the dominant eye is the eye less likely to lose fixation at the near point of convergence [13, 14], and the state of the extraocular muscles with their innervational patterns may play a role in determining motor dominance. Sensory dominance occurs when the perception of a stimulus presented to one eye dominates the other in retinal rivalry conditions [15]. Accurate determination of ocular dominance is especially useful in clinical decision making as it relates to monovision management of presbyopia [16–18] with intraocular lens implant in cataract surgery [19, 20], contact lenses [21, 22], or LASIK [23, 24].

For an eye with refractive error, incident light from a distant object focuses either in front of or behind the retina. Visual input-dependent feedback is generally considered the central mechanism for the development of refractive errors [25–27]. Since the two eyes do not always have the same amount of refractive error and ocular dominance reflects differential information processing through the visual pathways for the two eyes [1–3], it is interesting to explore the association between ocular dominance and refractive error asymmetry between the two eyes.

There have been studies addressing the question of the association of ocular dominance and refractive error. The results and conclusions have so far shown no convergence on any definite consensus. However, three different results can be delineated from the literature. Some studies with adult subjects have shown ocular dominance in the eye with greater myopia and axial length [4], while others have shown that the non-dominant eye has greater myopic and astigmatic errors [6–8]. The third group of results found no significant association between ocular dominance and mean spherical equivalent refractive errors [5, 9].

These previous studies have several limitations. Firstly, only two aspects of ocular dominance were measured. Specifically, the hole-in-the-card test was used to measure sighting dominance [4–7, 9], and convergence near-point test was used to measure motor dominance [10, 11]. The relationship between refractive error asymmetry and sensory dominance, which occurs when there is perceptual dominance of one eye over the other in retinal rivalry [15], has been largely ignored. The second limitation of past studies is that both the hole-in-the-card, that measures sighting dominance, and convergence near point test, that measures motor dominance are qualitative methods. They reveal eye dominance without quantitative description of the degree of relative dominance between the two eyes. Sensory dominance, on the other hand, could be precisely quantified through psychophysical methods.

This study articulates and provides a quantitative measure of the relationship between ocular sensory dominance and anisometropia in an effort to overcome the limitations of previous studies.

## Materials and Methods

### Subjects

A total of 219 subjects, 92 males and 127 females, were recruited from the clinic of Department of Optometry, Wenzhou Medical University. The ages ranged from 18–46 years with mean age at 27.44 ± 5.86 years.

To be included in the study, the subjects were required to have best corrected visual acuity (BCVA) of 20/20 or better at distance for each eye. Subjects were excluded from the study if any of the following conditions existed: latent hyperopia, history of strabismus or ptosis, any ocular surgery, amblyopia, keratoconus, glaucoma, retinal diseases, optic disc abnormalities, optic neuropathty, or other diseases that might affect BCVA, and apparent facial asymmetry that could be easily identified by visual evaluation.

The study was approved by the Ethics Board of the Wenzhou Medical University. Informed written consent was obtained from the subjects after explanation of the nature and possible consequences of the study. All procedures adhered to the Declaration of Helsinki of the World Medical Association.

### Refraction

Objective refraction measurements were obtained using the WAM 5500 autorefractor (Grand SEIKO Co. Ltd, Japan) without cycloplegia. During the measurement, the fixation target, a bright spot (20 cd/m^2^) on a dark computer screen, was located 6 meters in front of the eye. During testing, the non-tested eye was left open. Refractive errors (Spherical [S], Cylinder [C], axis [α]) were measured five times in each eye. Spherical equivalent (SE) and vector presentation of astigmatism J0 and J45 were calculated according to the following formulas: SE = S + C/2; J0 = (-C/2) * cos(2*α); J45 = (-C/2) * sin(2*α).

Emmetropia is defined as having refractive error less than 0.50 D, minus or plus. Myopia and hyperopia were defined as spherical equivalent refractive errors with absolute values greater than 0.5 D. Anisometropia was defined as interocular refractive error difference (IRED) with absolute value equal or greater than 0.75 D. A total of 176 myopic subjects were recruited, with 93 as non-anisometropic and 83 as anisometropic. A total of 43 hyperopic subjects were recruited, with 21 of them being non-anisometropic and 22 of them being anisometropic.

### Sighting dominance

Hole-in-the-card test was used to determine sighting dominance. In the test, the subject was asked to hold a card that has a 3-cm hole in the center, with both hands at arm’s length. The subjects viewed a target 6m away through the hole with both eyes open. Each eye was then covered in turn to identify the dominant one. When the dominant eye was covered, the target would disappear. When the non-dominant eye was covered, the target would not disappear.

### Motor dominance

Motor dominance was determined by the convergence near-point test. A subject was asked to fixate at the tip of a small stick that was moving toward the bridge of the nose, with both eyes open. During this process, an ophthalmologist observed whether divergence occurred in one of the eye. In cases that the deviation could be clearly identified, the eye that deviated first was taken as the non-dominant eye and the eye remained fixated was taken as the dominant one. Cases were classified as “undetermined” when it was difficult to decide which eye deviated first.

### Sensory eye dominance measurement

**Fig 1:**
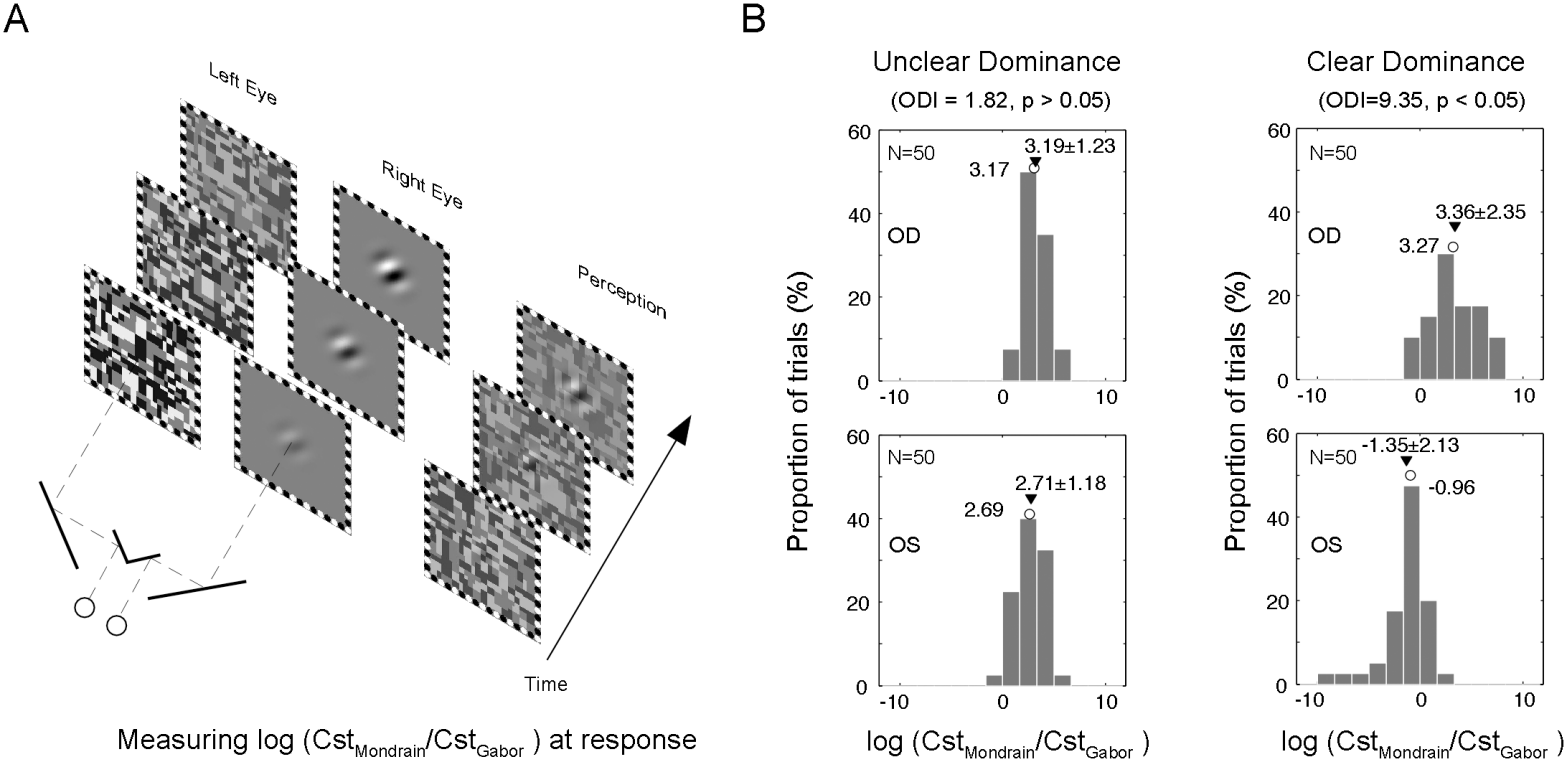
The method to measure ocular dominance index (ODI). (A) The log(Cst_Mondrian_/Cst_Gabor_) was calculated at response for each trial and each eye was tested 50 times. (B) T-test was used to compare the log ratios collected from the two eyes and the t-value was used as the ODI. Examples of subjects with unclear dominance (ODI < 2, left panel) and clear dominance (ODI ≥ 2, right panel) are shown here. Circles and triangles represent median and mean of the log ratios respectively.

The method used in this study to measure sensory dominance was a modified version of the method developed by Yang and Blake [28]. Briefly, stimuli were presented in the center of a CRT (1024 x 768 resolution; 100 Hz; Gamma corrected for linearity, Richardson Electronics) against a uniform background (mean luminance 50 cd/m^2^) and viewed at a distance of 60 cm with a chin rest. The dynamic Mondrian patterns subtended 4.3° x 4.3°, with individual elements extended 0.154°. The target stimulus was a Gabor patch tilted 45 degree toward either the right or the left (SF = 1c/d, spatial extension 1.9°). The black and white strokes that framed the Mondrian and Gabor were 0.33° in width and were used to help achieve binocular fusion. Mirrors were used to present the Mondrian and target stimuli dichoptically. Each eye exclusively viewed one of the two stimuli during a given trial. The eyes viewing the dynamic Mondrian and target stimuli were counterbalanced and randomized across trials (Fig 1). The experiment was programmed in commercial software (MatLab, Version 2012Rb; The MathWorks, Natick, MA, and the Psychophysics Toolbox, Version 3) [29, 30].

At the beginning of a trial, the measured eye viewed the target Gabor patch at 0% contrast and the fellow eye viewed a full contrast Mondrian pattern. During a trial, the contrast of the Gabor patch linearly increased at a rate of 1% every 100 ms, and the contrast of Mondrian patterns linearly decreased at the same rate. The subjects were tasked with reporting, by pressing one of two keys, when the obliquely oriented Gabor patches were detected. A trial terminated once the response was made. For each trial, the log ratio of Mondrian to Gabor’s contrasts [log(Cst_Mondrian_/Cst_Gabor_)] at the moment of response was computed. The higher the ratio, the greater the quantitative measure of the ocular sensory dominance of that eye. A high ratio means that the contrast detection threshold of the oriented Gabor patch needed to overcome the suppression imposed by the Mondrian stimulus on the other eye is low. In other words, the sensitivity of the eye with low contrast detection threshold, in overcoming Mondrian suppression, is high. All the subjects performed 10 practice trials before starting 50 experimental trials. It took about 15 minutes on average to complete the test for each subject.

T-test was used to compare the 50 values collected for each eye. T-value, which is the interocular difference in mean values normalized by the standard deviations of values from both eyes, was used as the ocular dominance index (ODI) to quantify a subject’s overall degree of ocular dominance. An ODI value of 2, which corresponds to a p value of 0.05 at a sample size of 50, was selected as the significance level. An ODI < 2, was regarded as having an unclear dominance. An example of a subject with unclear dominance is shown in the left panel of Fig 1B. On the other hand, a subject with an ODI ≥ 2 is regarded as having clear sensory dominance. An example of a subject with clear sensory dominance is shown in the right panel of Fig 1B, in which the right eye was the dominant eye.

All ocular dominance tests were conducted by the first author, an ophthalmologist, who was masked to the refractive status of the subjects.

### Data analyses and statistics

Data were entered into a spreadsheet and statistical analyses were performed with the Matlab software. Mean, median, and standard deviation were used for descriptive comparison. Two Wilcoxon rank-sum tests were performed. The first was to test for significant difference in refractive error between non-anisometropic and anisometropic subjects. While the second was to test for significant difference in ODI between non-anisometropic and anisometropic subjects (p < 0.05). Pearson’s r was used to calculate the correlation between the amplitude of anisometropia and ocular dominance index. The correlation coefficient was also tested for significance (p < 0.05). Sign-test was used to determine if the median value of interocular refractive error difference was significantly deviated from zero (p < 0.05). Kappa test was used to measure the concordance between three methods of ocular dominance.

## Results

### Refractive errors

The analyses of the results for all the myopic subjects revealed that the mean and SD of the spherical equivalent (SE) for the non-anisometropic sub-group was -3.82 ± 2.08 D, that of the anisometropic subjects was -3.92 ± 2.55 D (Table 1). There was no significant difference in the SE of both sub-groups, using the Wilcoxon rank sum test, p = 0.48. Ninety-three (93) myopic subjects were non-anisometropic, the mean and SD of the SE for their right and left eyes were, -3.85 ± 2.04 D, and -3.79 ± 2.12 D respectively. Wilcoxon rank-sum test also did not show any significant difference, p = 0.80. Eighty-three (83) of the myopic subjects were anisometropic, the mean and SD of the SE between their right and left eyes were -4.11 ± 2.47 D and -3.74 ± 2.63 D respectively. Although there was a larger interocular difference in the mean SE amongst the myopic subjects with anisometropia, when compared to those of the non-anisometropic subjects (1.96 ± 1.05 D), there was however no significant difference between the two sub-groups (Wilcoxon rank-sum test, p = 0.23).

**Table 1:** The refractive errors.

**Myopic Non-Aniso**					
N = 93	All eyes	OD	OS	p	abs(OD-OS)
SE (D)	-3.82 ± 2.08	-3.85 ± 2.04	-3.79 ± 2.12	0.80	0.33 ± 0.39
Astig (D)	-0.45 ± 0.56	-0.44 ± 0.54	-0.46 ± 0.58	0.10	0.21 ± 0.31
J0	-0.01 ± 0.17	-0.04 ± 0.19	0.01 ± 0.14	0.71	0.15 ± 0.25
J45	0.09 ± 0.30	0.08 ± 0.28	0.10 ± 0.33	0.92	0.11 ± 0.17
**Myopic Aniso**					
N = 83	All eyes	OD	OS	p	abs(OD-OS)
SE (D)	-3.92 ± 2.55	-4.11 ± 2.47	-3.74 ± 2.63	0.23	1.96 ± 1.05
Astig (D)	-0.78 ± 0.68	-0.77 ± 0.74	-0.80 ± 0.63	0.08	0.52 ± 0.62
J0	-0.00 ± 0.24	0.05 ± 0.22	-0.05 ± 0.25	0.90	0.28 ± 0.28
J45	0.20 ± 0.42	0.20 ± 0.44	0.20 ± 0.40	0.52	0.26 ± 0.30
**Hyperopic Non-aniso**					
N = 21	All eyes	OD	OS	p	abs(OD-OS)
SE (D)	1.08 ± 0.96	1.06 ± 0.96	1.11 ± 0.98	0.85	0.23 ± 0.21
Astig (D)	-0.35 ± 0.33	-0.36 ± 0.33	-0.34 ± 0.34	0.45	0.19 ± 0.19
J0	-0.03 ± 0.07	-0.04 ± 0.06	-0.03 ± 0.08	0.83	0.08 ± 0.08
J45	0.01 ± 0.23	-0.00 ± 0.24	0.02 ± 0.23	0.75	0.10 ± 0.09
**Hyperopic Aniso**					
N = 22	All eyes	OD	OS	p	abs(OD-OS)
SE (D)	1.42 ± 1.08	1.12 ± 1.04	1.71 ± 1.07	0.06	1.47 ± 0.68
Astig (D)	-0.69 ± 0.81	-0.67 ± 0.88	-0.72 ± 0.74	0.30	0.62 ± 0.59
J0	0.00 ± 0.22	0.01 ± 0.23	-0.00 ± 0.22	0.12	0.21 ± 0.21
J45	0.16 ± 0.46	0.10 ± 0.50	0.21 ± 0.42	0.82	0.25 ± 0.28

The results for all the hyperopic subjects revealed that the mean and SD of the SE for the non-anisometropic subjects was 1.08 ± 0.96 D, that of the anisometropic subjects was 1.42 ± 1.08 D (Table 1). Statistical analysis did not show any significant difference (Wilcoxon rank-sum test, p = 0.90). Twenty-one (21) of the hyperopic subjects were non-anisometropic, the mean and SD of the SE for right and left eyes were 1.06 ± 0.96 D, 1.11 ± 0.98 D respectively. There was no significant difference in mean SE between both eyes of subjects in this sub-group (Wilcoxon rank-sum test, p = 0.85). Twenty-two (22) of the hyperopic subjects were anisometropic. Their mean and SD of the SE between the right and left eyes were 1.12 ± 1.04 D and 1.71 ± 1.07 D. Here again we see a larger interocular difference in SE, but with no statistically significant difference (Wilcoxon rank-sum test, p = 0.06).

### Anisometropic subjects have stronger ocular dominance

**Fig 2:**
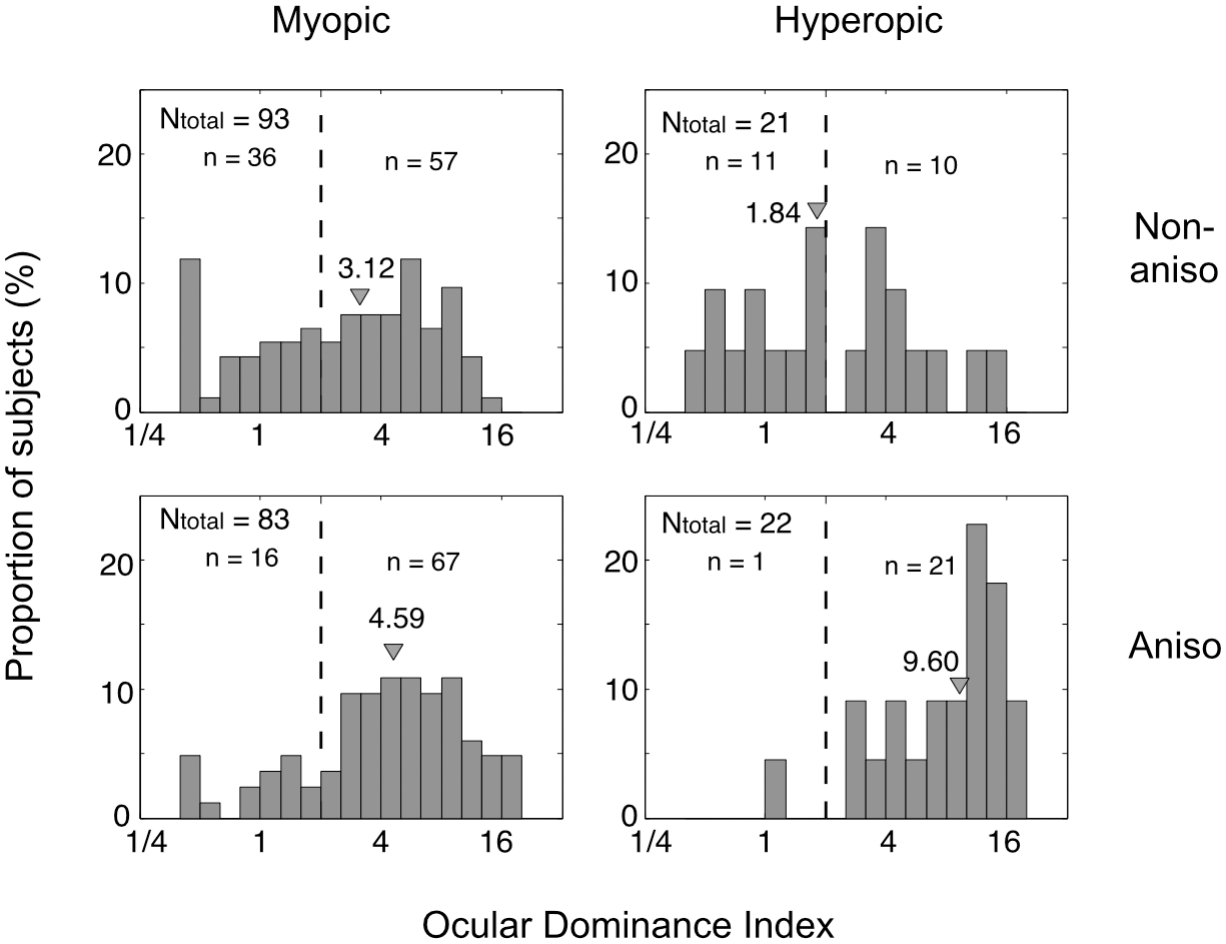
The distribution of ocular dominance index in myopic (left column) and hyperopic (right column) subjects. Top row: non-anisometropic subjects; bottom row: anisometropic subjects. Triangles represent median values.

In comparison to non-anisometropic subjects, anisometropic subjects had stronger ocular dominance. In myopic subjects, the median value of ODI was 4.59 for anisometropic subjects, which was significantly higher than that (3.12) of the non-anisometropic subjects (Wilcoxon rank-sum test, p < 0.01). For non-anisometropic subjects, 36 (38.7%) of them showed unclear dominance and 57 (61.3%) of them showed clear dominance. For anisometropic subjects, the proportion of subjects with unclear dominance was 19.3% (16) and that with clear dominance increased to 80.7% (67) (Fig 2, left column). In hyperopic subjects, the median value of ODI was 9.60 for the anisometropic subjects, which was significantly higher than that (1.84) of the non-anisometropic subjects (Wilcoxon rank-sum test, p < 0.01). For non-anisometropic subjects, 11 (52.4%) of them showed unclear dominance and 10 (47.6%) of them showed clear dominance. For anisometropic subjects, the proportion of subjects with unclear dominance was 4.5% (1) and those with clear dominance was 95.5% (21) (Fig 2, right column).

### The amount of anisometropia is correlated with the ocular dominance index

**Fig 3:**
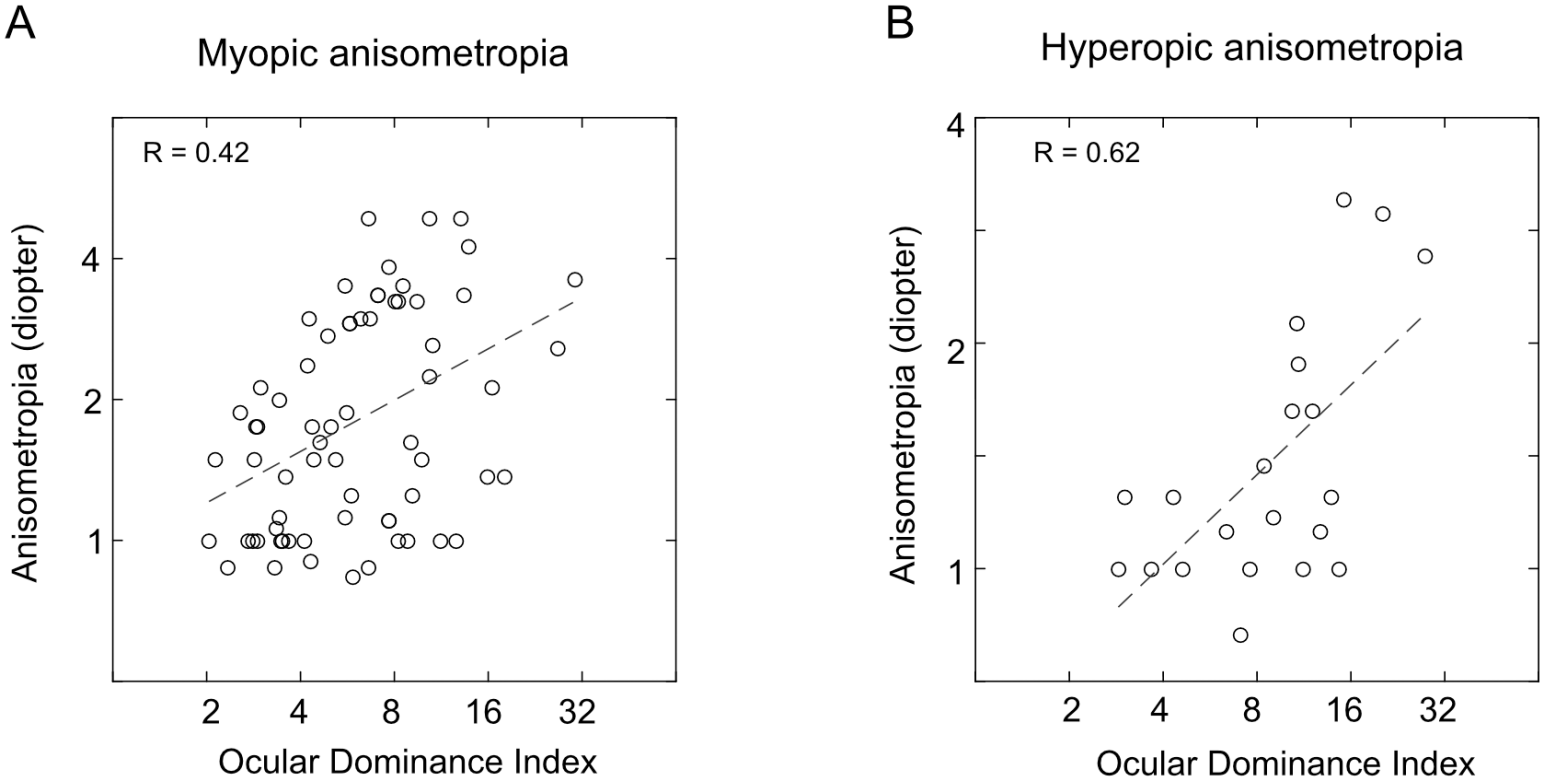
Correlation between the amplitude of anisometropia and ocular dominance index.

We further investigated whether stronger ocular dominance in anisometropic subjects was correlated with the degree of anisometropia. For this analysis, only those subjects with clear ocular dominance were included. In myopic anisometropic subjects (Fig 3A), there was a mild, but significant, (R = 0.42, p < 0.05) correlation between the value of ocular dominance index and the amount of anisometropia. In hyperopic anisometropic subjects (Fig 3B), the correlation between the value of ocular dominance index and amount of anisometropia was relatively higher (R = 0.62, p < 0.05).

### The dominant eyes are more myopic/ less hyperopic

**Fig 4:**
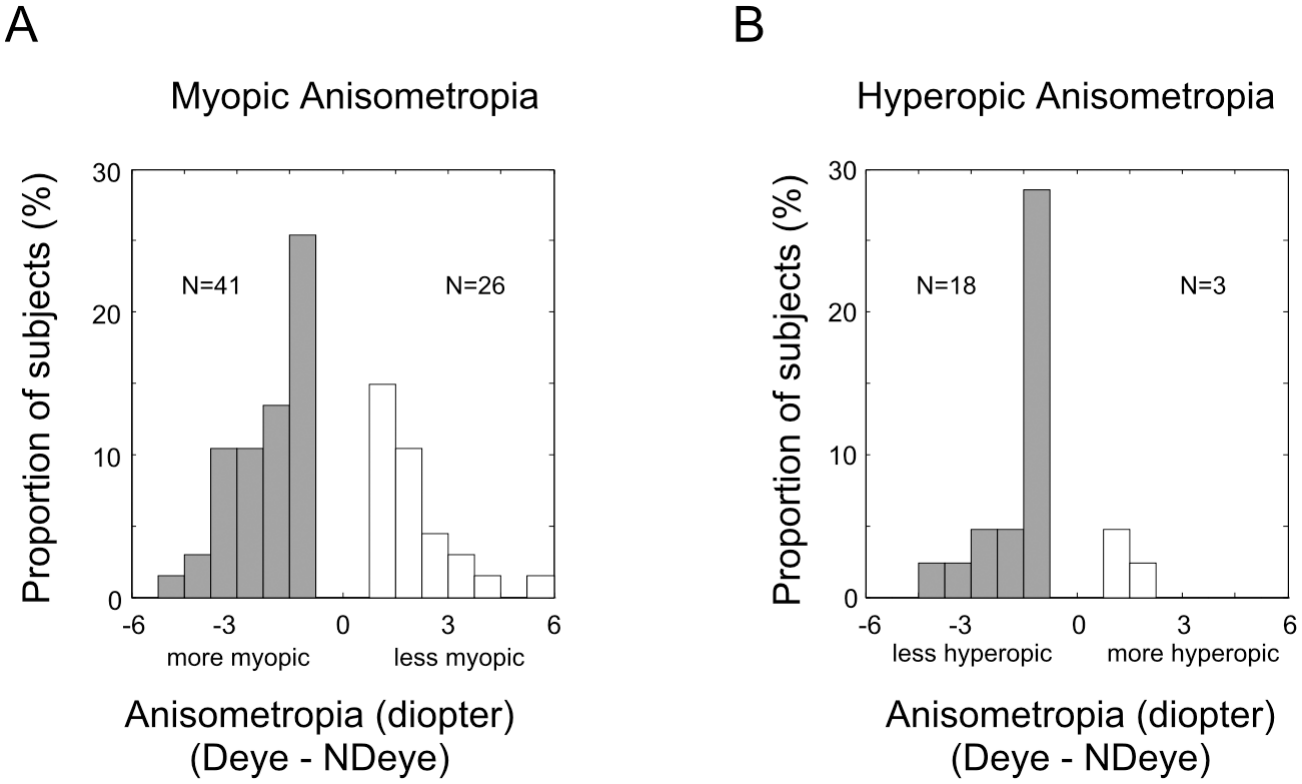
Dominant eyes were more myopic in myopic anisometropic subjects (A) and less hyperopic in hyperopic anisometropic subjects (B). The amplitude of anisometropia was calculated as the refractive error of the dominant eye minus the refractive error of the non-dominant eye.

Since the amplitude of anisometropia was correlated with the ocular dominance, it will be interesting to know whether the dominant eyes had relatively smaller refractive errors than the non-dominant eyes. In the hyperopic anisometropic subjects, the majority (85.7%, 18/21) of the dominant eyes were less hyperopic than the non-dominant eyes (Fig 4B). Quantitatively, the dominant eye was significantly less hyperopic (sign-test, p < 0.01). However, in the myopic anisometropic subjects, the majority (61.2%, 41/67) of the dominant eyes were more myopic than the non-dominant eyes (Fig 4A). Quantitatively, the dominant eyes were significantly more myopic (sign-test, p = 0.02).

### Three different methods accessing ocular dominance

We found a low concordance between three methods of measuring ocular dominance (Table 2). The concordance between sighting dominance and sensory dominance was rather low (kappa = 0.25, p < 0.01). The concordance between sighting dominance and motor dominance was relatively higher (kappa = 0.46, p < 0.01).

**Table 2:** Concordance among sighting, motor and sensory dominance.

Kappa = 0.46	Motor		Kappa = 0.25	Sensory		Kappa = 0.13	Sensory	
Sighting	R	L	Sighting	R	L	Motor	R	L
**R**	93	10	**R**	67	29	**R**	56	27
**L**	25	28	**L**	26	33	**L**	14	13
p<0.01			p<0.01			p = 0.2		

Among the myopic anisometropic subjects, based on sighting dominance, 50.6% of dominant eyes were more myopic (42/83), and the other 49.4% were less myopic (41/83). Based on motor dominance, 61.1% of the dominant eyes were more myopic (33/54). Among the hyperopic anisometropic subjects, based on sighting dominance, 77.2% of the dominant eyes were less hyperopic (17/22). Based on motor dominance, 68.8% of the dominant eyes were less hyperopic (11/16).

## Discussion

In this study, the relationship between ocular sensory dominance and anisometropia was explored. The anisometropic subjects had stronger ocular dominance with a higher proportion of them showing clear ocular dominance (Fig 2). In those anisometropic subjects with clear ocular dominance, the amplitude of the anisometropia was correlated with the strength of ocular dominance (ocular dominance index, Fig 3). More importantly, the dominant eyes were more myopic in myopic anisometropic subjects and less hyperopic in hyperopic anisometropic subjects (Fig 4). This finding may shed new lights on our understanding of the development of refractive error, particularly, anisometropia.

### The separation of clear from unclear dominance

One important feature of our study was the separation of the subjects with clear dominance from those with unclear dominance. In sighting dominance based on hole-in-the-card test [4, 6, 7], a subject was rarely labeled as showing unclear dominance. In our study, for every subject, the log ratio of Mondrian to Gabor’s contrasts at response was measured 50 times for each eye and ocular sensory dominance was decided based on the statistical comparison of values from each eye. This allowed us to separate the subjects into those with clear dominance from those with unclear dominance with high confidence [28]. It was only in the subjects with clear dominance that correlation between the strength of ocular dominance and the amplitude of anisometropia was clearly revealed (Fig 3). The lack of sensory dominance in subjects with unclear dominance would have confounded results of the relationship between ocular dominance and anisometropia if they were lumped with the data of subjects showing clear dominance.

### Quantifying the relationship between ocular dominance and amplitude of anisometropia

In our study, both the amplitude of the anisometropia and the degree of ocular dominance could be quantified. Therefore, the quantitative relationship between those two parameters could be plotted out as in Fig 3. Although the correlations were only mild, it was significant in both myopic anisometropic subjects and hyperopic anisometropic subjects. Sighting dominance and motor dominance are qualitative measures that can only tell which eye is the dominant one without showing the degree of dominance. The only quantification carried out in previous studies, [4, 6, 7], was usually on the degree of anisometropia. Subjects were usually separated into groups by the magnitude of anisometropia, and the presence or absence of ocular dominance tested without reference to the relative strength of the dominance. The significant association between ocular dominance and anisometropia were only found in the subjects with anisometropia beyond certain diopter. Cheng reported an association between anisometripia and ocular dominance, in subjects with myopic anisometropia larger than 1.75 D [4], while Ito et al. only found an association in subjects with anisometropia > 2 D (164 out of 3012 total subjects, 5%) [6]. Linke et al., on the other hand, found an association only in subjects with anisometropia > 2.5 D (278 out of total 10264 subjects, about 3%) [7].

### Comparison to previous studies

In this study, we reported that the dominant eyes were more myopic in myopic anisometropic subjects and less hyperopic in hyperopic anisometropic subjects. This finding contradicted some of the previous studies. Ito et al. reported that the dominant eyes were less myopic [6], and Linke et al. also reported that the dominant eyes were significantly less myopic [7]. However, our results were in agreement with Cheng’s finding, that in subjects with myopic anisometropia, the dominant eyes were more myopic than the non-dominant eyes [4]. The discrepancy in results could be partially explained by the fact that different methods were used to measure ocular dominance. Since sighting, motor, and sensory dominance may access different aspects of the ocular dominance, it is not surprising to see the low concordance among the three methods and contradiction between our study and some of the previous studies.

### Speculation on ocular dominance and the development of anisometropia

Refractive development usually starts with both eyes in hyperopic status and the growth of the eyeball is guided by the feedback from both retina and visual brain, where binocular information is processed [25–27]. Ocular dominance measures relative sensitivity of the visual brain to the signals sent from each eye under binocular viewing conditions, with the sensory dominant eye presumed to have greater access to the visual brain. One possible speculation is that greater sensitivity leads to greater growth rate. In that scenario, for a subject with unclear dominance, the equal sensitivity of the two eyes would promote equal eye growth. For a subject with clear dominance, the dominant eye, with higher sensitivity, would grow faster and the non-dominant eye would grow slower. During the emmetropization stage, the dominant eye is less hyperopic and the non-dominant eye is more hyperopic. If the development continues into the myopization stage, the dominant eye tends to be more myopic and the non-dominant eye is less myopic. However, this speculation should be treated with caution. First, in our myopic anisometropic subjects, not all of the dominant eyes were more myopic. In 38.8% of the subjects, the non-dominant eyes were more myopic. Second, the sample size for the hyperopic anisometropia was relatively small. Third, it is not clear whether the dominant eye remains the same or switches between viewing distance and near. Our lab is currently testing this issue. Fourth, a cross-sectional design was used in this study and therefore could not address one of the pertinent questions of which came first, anisometropia or ocular dominance? An appropriately designed longitudinal study that shows the temporal relationship between ocular sensory dominance and anisometropia, in the developmental stages of children, is needed to answer this question. The protocol for such a study should include, assessing ocular dominance and refractive errors at an early age, preferably before the age of 3 years before ocular dominance is established. The baseline measurements should then be followed up with routine measures on a quarterly basis until their teenage years. This will map out a developmental curve of ocular dominance and anisometropia as a function of age. Such a curve will clearly demonstrate whether the onset of ocular dominance precedes the emergence of anisometropia or vice-versa. The study will also show whether the dominant eye becomes less hyperopic or more myopic, and if the degree of anisometropia correlates with the degree of ocular dominance. Data collected from such a longitudinal study may help to elucidate the causality, particularly in those children with clear sensory dominance and large magnitude of anisometropia.

## Conclusions

We conclude that anisometropic subjects have stronger ocular dominance, that the amplitude of the anisometropia is correlated with the strength of ocular dominance, and that the dominant eyes are more myopic in myopic anisometropic subjects and less hyperopic in hyperopic anisometropic subjects.
